# Repercussions of COVID-19 in hemodialysis patients: a systematic review

**DOI:** 10.15649/cuidarte.2695

**Published:** 2023-03-31

**Authors:** Tahissa Frota-Cavalcante, Raphaella Castro-Jansen, José Erivelton de Souza-Maciel-Ferreira, Cristefania Meirú-de-Lima, Huana Carolina Candido-Morais, Rafaella Pessoa-Moreira

**Affiliations:** 1 . University of International Integration of Afro-Brazilian Lusophony, Brazil. Email: tahissa@unilab.edu.br University of International Integration of Afro-Brazilian Lusophony Brazil tahissa@unilab.edu.br; 2 . University of International Integration of Afro-Brazilian Lusophony, Brazil. Email: raphaella.jansen@gmail.com University of International Integration of Afro-Brazilian Lusophony Brazil raphaella.jansen@gmail.com; 3 . University of International Integration of Afro-Brazilian Lusophony, Brazil. Email: eriveltonsmf@gmail.com University of International Integration of Afro-Brazilian Lusophony Brazil eriveltonsmf@gmail.com; 4 . University of International Integration of Afro-Brazilian Lusophony, Brazil. Email: crismeiru@gmail.com University of International Integration of Afro-Brazilian Lusophony Brazil crismeiru@gmail.com; 5 . University of International Integration of Afro-Brazilian Lusophony, Brazil. Email: huanacarolina@unilab.edu.br University of International Integration of Afro-Brazilian Lusophony Brazil huanacarolina@unilab.edu.br; 6 . University of International Integration of Afro-Brazilian Lusophony, Brazil. Email: rafaellapessoa@unilab.edu.br University of International Integration of Afro-Brazilian Lusophony Brazil rafaellapessoa@unilab.edu.br

**Keywords:** SARS-CoV-2, COVID-19, Hemodialysis, Renal Insufficiency, Chronic, Nursing., SARS-CoV-2, COVID-19, Diálisis Renal, Insuficiencia Renal Crónica, Enfermería., SARS-CoV-2, COVID-19, Diálise Renal, Insuficiencia Renal Crónica, Enfermagem.

## Abstract

**Introdution:**

The immunosuppressive state of patients with CKD increases their risk of developing poor clinical outcomes if they acquire COVID-19 infection.

**Objective:**

To identify the scientific evidence about the repercussions of COVID-19 in hemodialysis patients.

**Materials and Methods:**

A systematic review was conducted in this study. The databases Cochrane Library, Web of Science, Science Direct, PubMed, and Virtual Health Library were searched to identify relevant studies. The methodological quality of the studies was assessed using the adapted Downs and Black checklist. The review adhered to the PRISMA guidelines.

**Results:**

A total of 16 articles were included after the screening process. All articles had a methodological quality higher than 66.8%. The most common repercussions of COVID-19 in hemodialysis patients were the increased mortality rate (75%), development of typical signs and symptoms of the disease such as fever, cough, dyspnea, and fatigue (68.75%), lymphopenia (68.75%), progression to severe acute respiratory syndrome (56.25%), need for mechanical ventilation (50%), and admission to intensive (50%).

**Conclusions:**

The hemodialysis patients are more susceptible to COVID-19 infection and, when infected by SARS-CoV-2, these patients have more adverse clinical outcomes, more serious diseases, higher mortality, and worse prognosis than the general population. The repercussions of COVID-19 in hemodialysis patients reveal a need for preventive nursing care in hemodialysis clinics.

## Resumen

**Introducción:**

El estado de inmunosupresión de los pacientes con enfermedad renal crónica (ERC) aumenta su riesgo de obtener resultados clínicos desfavorables si llegaran a contraer COVID-19.

**Objetivo:**

identificar la evidencia científica acerca de las repercusiones que tiene el COVID-19 en los pacientes en hemodiálisis.

**Materiales y métodos:**

Se realizó una revisión sistemática en este estudio. Se hizo una búsqueda en las bases de datos Cochrane Library, Web of Science, Science Direct, PubMed y Virtual Health Library para identificar estudios relevantes. La calidad metodológica de los estudios se evaluó mediante la lista de chequeo adaptada por Downs y Black. La revisión siguió los lineamientos de la declaración PRISMA.

**Resultados:**

Tras el proceso de selección se incluyeron un total de 16 artículos en la revisión. Todos los artículos obtuvieron una calidad metodológica superior a 66,8%. Las repercusiones más comunes del COVID-19 en los pacientes en hemodiálisis fueron el aumento de la tasa de mortalidad (75%), el desarrollo de signos y síntomas típicos de la enfermedad como fiebre, tos, disnvvvea y fatiga (68,75%), linfopenia (68,75%), progresión a un síndrome respiratorio agudo grave (56,25%), necesidad de ventilación mecánica (50%) e ingreso a cuidados intensivos (50%).

**Conclusiones:**

Los pacientes en hemodiálisis son más susceptibles a contraer COVID-19 y, cuando contraen el SARS-CoV-2, tienen resultados clínicos más adversos, enfermedades más graves, mayor mortalidad y peor pronóstico que la población general.

## Resumo

**Introdujo:**

O estado imunossupressor dos pacientes com CKD aumenta seu risco de desenvolver maus resultados clínicos se eles adquirirem a infecgáo COVID-19.

**Objetivo:**

Identificar as evidencias científicas sobre as repercussoes da COVID-19 em pacientes com hemodiálise.

**Materiais e Métodos:**

Uma revisáo sistemática foi conduzida neste estudo. As bases de dados Cochrane Library, Web of Science, Science Direct, PubMed e Virtual Health Library foram pesquisadas para identificar estudos relevantes. A qualidade metodológica dos estudos foi avaliada utilizando a lista de verificado Downs e Black adaptada. A revisáo seguiu as diretrizes do PRISMA.

**Resultados:**

Um total de 16 artigos foram incluídos após o processo de triagem. Todos os artigos tinham uma qualidade metodológica superior a 66,8%. As repercussoes mais comuns da COVID-19 em pacientes de hemodiálise foram o aumento da taxa de mortalidade (75%), desenvolvimento de sinais e sintomas típicos da doenga como febre, tosse, dispnéia e fadiga (68,75%), linfopenia (68,7v5%), progressáo para síndrome respiratória aguda grave (56,25%), necessidade de ventilado mecánica (50%) e admissáo a intensivo (50%).

**Conclusóes:**

Os pacientes em hemodiálise sáo mais suscetíveis a infecgáo por COVID-19 e, quando infectados pela SRA-CoV-2, estes pacientes tem resultados clínicos mais adversos, doengas mais graves, maior mortalidade e pior prognóstico do que a populado em geral. As repercussoes da COVID-19 em pacientes de hemodiálise revelam uma necessidade de cuidados preventivos de enfermagem em clínicas de hemodiálise.

## Introduction

COVID-19 is a highly contagious disease, whose cases range from mild to severe. It is considered a systemic disease that affects multiple organs and the kidneys are one of the most common organs affected by SARS-CoV-2^1^. Specific groups of patients, such as those with chronic non-communicable diseases, are more likely to develop severe acute respiratory syndrome (SARS) ^(^^1^.

Patients with chronic kidney disease (CKD) are among the population considered at risk for severe COVID-19, especially those with associated comorbidities including diabetes mellitus and systemic arterial hypertension^2^. In 2017, the global estimated prevalence of CKD was 9.1%^3^. Hemodialysis stands out among the treatment modalities available for patients with CKD, being adopted by 92% of patients with this condition.

Patients with CKD are exposed to the virus more frequently than the general population, as they need to go out for hemodialysis sessions^2^. Given the impossibility of maintaining social isolation and abandoning treatment (essential for the maintenance of life), patients with CKD are more likely to become infected and develop severe SARS-CoV-2 infection^4^. In addition to the above, hemodialysis requires intensive care due to the possibility of clinical complications.

Since nurses are at the front line fighting the COVID-19 pandemic, they occupy a prominent position in the care of patients affected by this infection. These professionals also play an essential role in the hemodialysis service. With the advent of the COVID-19 pandemic, and due to the vulnerability of hemodialysis patients, nurses' responsibilities have become greater as it is necessary to coordinate efforts to prevent and control the transmission of the coronavirus^5^.

Issues related to signs, symptoms, and clinical outcomes prevalent in this population need to be elucidated, as few studies address the effects of COVID-19 in hemodialysis patients. Therefore, the following research question arose: What are the repercussions of COVID-19 in patients undergoing hemodialysis? This study's findings may be relevant to understanding the effects of COVID-19 infection in hemodialysis patients, facilitating the decision-making of nurses fighting this pandemic.

The aim of this systematic review was to identify the scientific evidence about the repercussions of COVID-19 in hemodialysis patients.

## Materials and Methods

A systematic review of the literature was conducted. The review adhered to the Preferred Reporting Items for Systematic Reviews and Meta-Analyses (PRISMA) guidelines^6^. The review protocol was registered in PROSPERO. The research question for the systematic review was developed based on the PECOS framework^7^ (Table 1). The research question was: What are the repercussions of COVID-19 in hemodialysis patients?

**Table 1:** Research question development process using the PECOS framework.

Element definition	Acronym	Description
Population	P	Patients undergoing hemodialysis treatment
Exposure	E	COVID-19
Comparator	C	Not applicablea
Outcome	O	Repercussions of COVID-19 in hemodialysis patients
Study design	S	Observationalb and experimental research

*aNot applicable, as the review was not restricted to clinical trials; bCohort, case-control, cross-sectional, qualitative, and quantitative studies.Source: Authors, 2021.*

The inclusion criteria were primary studies, available online, published from 2019 to 2021, in any language, and studies addressing the repercussions or impacts of COVID-19 in hemodialysis patients. The literature search was carried out in February 2021 through the electronic databases: Cochrane Library, Web of Science, and Science Direct. In addition, PubMed and the Virtual Health Library were searched using direct access.

The search strategies were determined using the MeSH and DeCS (Portuguese Health Sciences Descriptors) terms presented in Table 2.

**Table 2:** Database search strategies.

Database	Search strategies
PubMed	(SARS-CoV-2) AND ("Haemodialysis")
Virtual Health Library Cochrane	(SARS-CoV-2) AND (Hemodiálise) AND complicates
Web of Science	(SARS-CoV-2) AND (Haemodialysis)
Science Direct	(SARS-CoV-2) AND (Haemodialysis)
	(covid-19) AND (Haemodialysis)

The eligibility of the articles took place in two stages. In the first stage, two independent reviewers evaluated the titles and abstracts and identified potentially eligible studies. In the second stage, the full texts were read to confirm eligibility. Any disagreement was resolved by consensus or by a third reviewer. Duplicates were identified and removed through the Mendeley Desktop platform version 1.19.4. Data related to findings reported in the present manuscript were saved as a dataset (Mendeley Data8).

The methodological quality of the studies was assessed using the adapted Downs and Black checklist, which allows the assessment of the risk of bias in randomized and non-randomized studies^9^. In the present study, questions 4, 8, 12, 13, 14, 15, 19, 23, 24, and 27 were excluded because they refer to studies with a randomized clinical trial design, not retrieved in this research. Thus, 17 items from the original scale were used. Previous studies judged their articles according to the following categories: low methodological quality (<33.3%), moderate methodological quality (33.4-66.7%), and high methodological quality (>66.8%10). Thus, we followed this same categorization in the present review.

The articles were assessed for quality according to criteria defined by the Oxford Center for Evidence Based Medicine^11^. As we included studies with different methodological designs, meta-analysis was not performed, and the data synthesis was qualitative.

## Results

**Figure 1:**
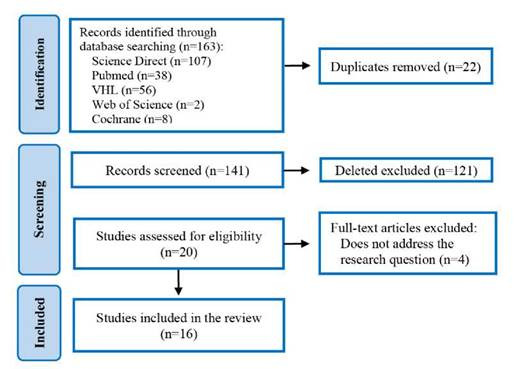
PRISMA Flowchart of the search strategy.

Initially, the research enabled the inclusion of 163 studies, and of these, 22 duplicates were excluded. After the full-text screening, 125 articles were excluded for not meeting all the inclusion criteria. At the end of the selection process, 16 studies composed the final sample (Figure 1).

Of the 16 articles analyzed, 13 (81.25%) were cohort studies, two (12.5%) were case-control studies, and one was a cross-sectional study. As for the quality assessment, all articles had a percentage above 66.8% (high methodological quality). Regarding the level of evidence, studies classified as 2B (cohort studies) predominated (n=13; 81.25%). Table 3 shows detailed information of the articles included in the final review sample.

**Table 3:** Synthesis of the articles included in the systematic review.

ID	Author and year	Location and sample	Article title	Design and Evidence level	Downs and Black score
A1	Ossareh et al./ 2020^12^	Iran	Role of Screening for COVID-19 in He modialysis Wards, Results of a Single Center Study.	Cohort	83%
A2	Zhang et al./ 2020^13^	China	Clinical characteristics of 31 hemo dialysis patients with 2019 novel coro navirus: a retrospective study.	Level 2B	94%
A3	Alberici et al./ 2020^14^	Italy	A report from the Brescia Renal CO VID Task Force on the clinical charac teristics and short-term outcome of he modialysis patients with SARS-CoV-2 infection.	Cohort	83%
ID	Author and year	Location and sample	Article title	Design and Evidence level	Downs and Black score
A4	Goicoechea et al./ 2020^15^	Spain	COVID-19: clinical course and outcomes of 36 hemodialysis patients in Spain.	Level 2B	94%
A5	Wu et al./ 2020^5^	China	Clinical Features of Maintenance Hemodialysis Patients with 2019 Novel Coronavirus-Infected Pneumonia in Wuhan, China.	Cohort	94%
A6	Sánchez-Pérez et al./ 2020^16^	Valencia	Results of a healthcare organization model for COVID-19 in hemodialysis in a tertiary hospital and its subsidized centres.	Level 2B	72%
A7	Ebana et al./ 2021^17^	Cameroon	Epidemiologic and clinical profile, 90 days survival of incident end stage renal	Cohort	94%
A8	Savino et al./ 2020^18^	United King dom n=2.385	Sociodemographic features and mortality of individuals on haemo dialysis treatment who test positive for SARS-CoV-2: A UK Renal Registry data analysis.	Cohort	72%
A9	Albalate et al./ 2020^19^	Madrid	Sociodemographic features and mor tality of individuals on haemodialysis treatment who test positive for SARS- CoV-2: A UK Renal Registry data analysis.	Case-control	67%
A10	Giaime et al./ 2020^20^	France	High prevalence of asymptomatic CO- VID-19 in hemodialysis. Daily learning during first month of COVID-19 pan demic.	Level 3B	94%
A11	Ozturk et al./ 2020^21^	Turkey	Hydroxychloroquine and azithromycin tolerance in haemodialysis patients du ring COVID-19 infection.	Cohort	94%
A12	Bell et al./ 2020^22^	Scotland	Mortality analysis of COVID-19 infection in chronic kidney disease, haemodialysis and renal transplant patients compared with patients wi thout kidney disease: a nationwide analysis from Turkey.	Level 2B	100%
A13	Cunha et al./ 2020^23^	Spain	COVID-19 in patients with chronic kidney replacement therapy and kid ney transplant recipients in Scotland: findings and experience from the Sco ttish renal registry.	Cohort	78%
A14	Keller et al./ 2020^24^	France	The Spectrum of Clinical and Serological Features of	Level 2B	78%
A15	Trivedi et al./ 2020^25^	India	COVID-19 in Urban Hemodialysis Patients.	Cohort	67%
A16	Hendra et al./ 2021^26^	United Kign- dom	Impact of first-wave Coronavi rus disease 2019 infection in patients on haemodialysis in Alsace: the observational covidial study.	Level 2B	89%

**Figure 2:**
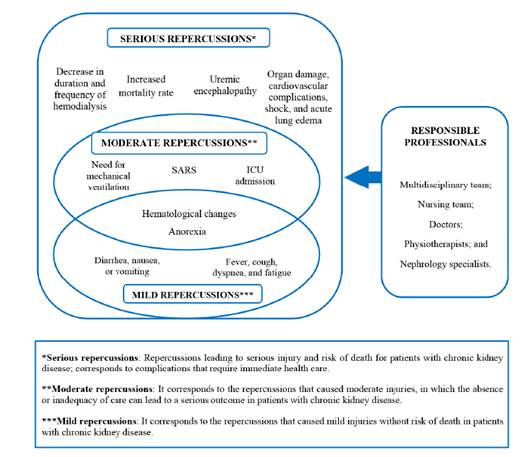
Repercussions of COVID-19 in hemodialysis patients.

The main results of the included studies show that laboratory abnormalities and poor clinical outcomes are common in hemodialysis patients with COVID-19. Table 4 and Figure 2 summarizes the impacts and repercussions of COVID-19 in hemodialysis patients

**Table 4:** Impacts of COVID-19 in hemodialysis patients.

Complications	Impact of COVID-19 in hemodialysis patients	Article ID	Frequency of studies
General	Increased mortality rate	A3-A5, A7-A10, A12-A16	75% (n=12)
	Need for mechanical ventilation	A4, A5, A10-A12, A14-A16	50% (n=8)
	ICU admission Organ damage, cardiovascular	A4, A6, A10-A14, A16	50% (n=8)
	complications, shock, and acute lung edema	A2, A5, A7	18.75% (n=3)
	Decrease in duration and frequency of hemodialysis	A2, A15	12.5% (n=2)
Respiratory	Development of signs and symptoms: fever, cough, dyspnea, and fatigue	A1-A4, A6, A8-A10, A13-A15	68.75% (n=11)
	Severe forms of COVID-19 infection and SARS	A2, A3, A5, A6, A11, A13-A16	56.25% (n=9)
Hematologic	Lymphopenia	A1, A2, A4, A5, A7, A9-A11, A13, A14, A16	68.75% (n=11)
	Albumin or glucose decrease	A2, A4, A11, A16	25% (n=4)
	Elevated D-dimer levels	A2, A5, A13, A16	25% (n=4)
	Elevated ferritin levels	A10, A11, A13	18.75% (n=3)
	Elevated troponin T levels	A10, A13, A16	18.75% (n=3)
	Elevated phosphorus levels	A2, A5	12.5% (n=2)
Gastrointestinal	Gastrointestinal symptoms (diarrhea, nausea, or vomiting)	A4, A6, A13, A14, A16	31.25% (n=5)
	Anorexia.	A5, A14	12.5% (n=2)
Neurological	Uremic encephalopathy	A7	6.25% (n=1)

It was observed that the most cited repercussions of COVID-19 in hemodialysis patients are related to general complications, such as high mortality (75%), respiratory complications and exacerbation of symptoms such as cough, dyspnea, fever and fatigue (68.75%), and progression to SARS (56.25%). Below is a graphical outline of the repercussions of COVID-19 on hemodialysis patients, classified according to their severity (Figure 2)

## Discussion

In general terms, it was observed that the repercussions of the disease were similar across the studies, and they are related to the immunodeficiency status resulting from the kidney disease itself.

Studies indicate that hemodialysis patients are more likely to develop severe COVID-19 infection and SARS and that hemodialysis patients had more severe infection rates than the general population^13^. The uremic state that affects patients with CKD and the immunosuppression explain the high incidence of COVID-19 infection in these patients^12^^,^^19^.

The typical symptoms of COVID-19 presented by hemodialysis patients are the same as the general population and include fever, cough, dyspnea, and fatigue. However, research has pointed out that hemodialysis patients infected with COVID-19 may not have a fever, as the incidence of hypothermia in this population is frequent^27^. Previous testing for COVID-19 through an individualized approach in hemodialysis patients can be a relevant strategy to optimize diagnostic accuracy and adequate control of virus transmissibility among patients, healthcare staff, and other contacts.

Given the complications caused by COVID-19, many patients on hemodialysis were admitted to intensive care units and needed mechanical ventilation. Another respiratory complication evidenced is acute lung edema^17^. In addition to SARS, a case-control study concluded that hemodialysis patients were more likely to develop shock and acute cardiac arrest^5^.

In this context of hospitalization and admission of these patients in intensive care units, nursing care in the hospital environment must be provided efficiently and integrated to the multidisciplinary team. A series of nursing actions must be carried out to promote the patients' safety, such as infection control measures, oral hygiene, aspiration of secretions, and vital signs monitoring. It is essential that the assistance of health professionals, especially nurses, in whatever environment the patient is in, is provided holistically and systematically to ensure correct and efficient interventions that improve patient outcomes.

Concerning gastrointestinal manifestations, studies show that diarrhea, nausea, vomiting, and respiratory symptoms were more intense and frequent in hemodialysis patients with COVID-19^28^^).^ In this scenario, the role of nurses working in outpatient clinics and hemodialysis clinics should be directed towards carrying out a holistic assessment of the patient, seeking to identify the phenomena resulting from hemodialysis therapy or COVID-19.

Among hematologic complications, several authors warn that lymphopenia is a prevalent laboratory finding in COVID-19 patients, being significantly more frequent in CKD patients on hemodialysis. A study comparing blood test results before and after COVID-19 infection showed that 51.6% of patients had a significant decrease in lymphocyte count^13^^,^^15^^,^^26^. Lymphopenia should be considered part of the diagnosis and a risk marker, especially in hemodialysis patients.

Another significant blood abnormality found was increased D-dimer, a marker of vascular damage. This abnormality was detected in 31 hemodialysis patients with COVID-19 in Wuhan, China, where 96.8% had increased D-dimer^13^. D-dimer is a marker that is elevated in hemodialysis patients. However, this increase was approximately three times greater than the normal values (68-494 pg) in hemodialysis patients with COVID-19^23^.

Decreased albumin and glucose levels were laboratory findings described in many studies^13^^,^^15^^,^^21^^,^^26^. Existing evidence suggests that the inflammatory state, evidenced by elevated C-reactive protein levels, can suppress albumin synthesis in hemodialysis patients^29^. In this context, care nurses and nephrologists need to recognize early predictive signs of complications presented by chronic renal patients to provide quick, timely, and adequate care.

Some authors also describe that hemodialysis patients with COVID-19 had higher values of phosphorus than the general population^5^^,^^13^. This is due to the excessive intake of foods rich in phosphorus and the low efficiency in removing phosphorus from plasma by the dialysis method. The role of nurses in educating patients on eating a balanced diet is important and enables the establishment of effective communication with patients and family members, facilitating treatment adherence and, consequently, a better quality of life.

An analysis in Peru compared patients hospitalized with COVID-19 at various stages of kidney disease and found that ferritin levels were significantly higher in those on hemodialysis^23^. For all this, ferritin can help screen COVID-19 in asymptomatic or non-symptomatic hemodialysis patients.

Researchers conducted a cohort study with hemodialysis patients and identified that troponin T levels were high in those with COVID-1920. Even without the association with cardiac lesions, high troponin levels indicate a worse prognosis26. In this context, when evaluating a patient with CKD, nurses should be aware of increased values of basal cardiac troponin T or I.

A study found that of 24 patients with COVID-19, 83.4% developed uremic encephalopathy^17^. As the nursing team is responsible for providing direct and indirect care to hemodialysis patients, it is up to these professionals to identify complications and initially assess the patients' clinical status to establish appropriate multidisciplinary conduct.

Although risk factors for COVID-19 are similar to the general population, patients on hemodialysis have a higher mortality rate. In an observational study in Spain, the authors found that hemodialysis patients with COVID-19 who died had lymphopenia, increased C-reactive protein and lactate dehydrogenase, and a longer time on hemodialysis treatment^15^. Given the above, it is essential to identify early risk factors and provide timely treatment for critical cases to reduce mortality and hospitalization in this highly vulnerable group.

In addition to the clinical effects, the repercussions of COVID-19 also influenced the dialysis treatment of patients on chronic dialysis regimens. Surveys show that a third of patients missed regular dialysis sessions^13^^,^^25^. In this sense, such treatment must be maintained during the pandemic, which involves care and procedures adopted by patients, health professionals, dialysis center managers, and health authorities.

As professionals who work on the front line in hospital, outpatient and clinical services, nurses have an essential role in the care of critically ill patients, as they carry out the planning of actions and the execution of work processes essential to quality patient care. The nursing team is a reference in the hemodialysis unit, providing direct care to patients who need this therapeutic modality to survive.

It is necessary to constantly update general nurses and specialists regarding the specifics of chronic renal patients undergoing renal replacement therapy. It is also recommended that the dialysis team always be aware of the signs and symptoms of COVID-19, in addition to managing transmission control. Although the pandemic scenario remains, it is important to maintain this treatment, considering the risks and benefits highlighted and discussed in the present study.

Among the study's limitations, it was observed that some studies were based on experiences developed in some institutions, which may differ from the reality of other locations. In addition, the small sample size of some studies is a limitation. In this sense, the results of this review highlighted the need for cross-sectional and cohort studies with larger sample sizes on hemodialysis patients with COVID-19 to understand better the repercussions of COVID-19 in this population.

It is believed that the information from this study may help health professionals in identifying potential risks to renal patients affected by SARS-CoV-2, in addition to facilitating the recognition of clinical priorities and potential supporting treatment, especially in those with a high risk of death.

## Conclussions

People with CKD affected by SARS-CoV-2 have a worse prognosis and a high mortality rate. The major repercussions of COVID-19 in patients with CKD undergoing hemodialysis are general, such as the need for ICU admission, mechanical ventilation, and high mortality rate. In addition, the damage of target organs such as the heart, lungs, and shock has been described. The main complications found through the review divided by bodily systems are SARS (respiratory), uremic encephalopathy (neurological), nausea, vomiting, and anorexia (gastrointestinal), and blood abnormalities. Therefore, hemodialysis patients are more susceptible to severe COVID-19, especially those with associated comorbidities.
